# Type 2 Diabetes Education and Support in a Virtual Environment: A Secondary Analysis of Synchronously Exchanged Social Interaction and Support

**DOI:** 10.2196/jmir.9390

**Published:** 2018-02-21

**Authors:** Allison A Lewinski, Ruth A Anderson, Allison A Vorderstrasse, Edwin B Fisher, Wei Pan, Constance M Johnson

**Affiliations:** ^1^ Durham Center for Health Services Research in Primary Care Durham Veterans Affairs Health Care System Durham, NC United States; ^2^ School of Nursing University of North Carolina at Chapel Hill Chapel Hill, NC United States; ^3^ Rory Meyers College of Nursing New York University New York, NY United States; ^4^ Gillings School of Global Public Health University of North Carolina at Chapel Hill Chapel Hill, NC United States; ^5^ Peers for Progress Gillings School of Global Public Health University of North Carolina at Chapel Hill Chapel Hill, NC United States; ^6^ School of Nursing Duke University Durham, NC United States; ^7^ Cizik School of Nursing The University of Texas Health Science Center at Houston Houston, TX United States

**Keywords:** type 2 diabetes, social interaction, self-management, virtual reality, social support

## Abstract

**Background:**

Virtual environments (VEs) facilitate interaction and support among individuals with chronic illness, yet the characteristics of these VE interactions remain unknown.

**Objective:**

The objective of this study was to describe social interaction and support among individuals with type 2 diabetes (T2D) who interacted in a VE.

**Methods:**

Data included VE-mediated synchronous conversations and text-chat and asynchronous emails and discussion board posts from a study that facilitated interaction among individuals with T2D and diabetes educators (N=24) in 2 types of sessions: education and support.

**Results:**

VE interactions consisted of communication techniques (how individuals interact in the VE), expressions of self-management (T2D-related topics), depth (personalization of topics), and breadth (number of topics discussed). Individuals exchanged support more often in the education (723/1170, 61.79%) than in the support (406/1170, 34.70%) sessions or outside session time (41/1170, 3.50%). Of all support exchanges, 535/1170 (45.73%) were informational, 377/1170 (32.22%) were emotional, 217/1170 (18.55%) were appraisal, and 41/1170 (3.50%) were instrumental. When comparing session types, education sessions predominately provided informational support (357/723, 49.4%), and the support sessions predominately provided emotional (159/406, 39.2%) and informational (159/406, 39.2%) support.

**Conclusions:**

VE-mediated interactions resemble those in face-to-face environments, as individuals in VEs engage in bidirectional exchanges with others to obtain self-management education and support. Similar to face-to-face environments, individuals in the VE revealed personal information, sought information, and exchanged support during the moderated education sessions and unstructured support sessions. With this versatility, VEs are able to contribute substantially to support for those with diabetes and, very likely, other chronic diseases.

## Introduction

Virtual environments (VEs) are one way to provide education and support to individuals with chronic illness. These 3D computer-generated replications of real-world settings foster interaction among individuals who interact as avatars (eg, computer-generated representations of humans) [1,2]. A VE imitates real-world interactions because an individual experiences *presence* (eg, feeling one is in the VE) and *copresence* (eg, feeling others are in the VE) [1,3]. These feelings of presence and copresence may accurately replicate real-world group interactions among individuals [4,5], because these interactions are bidirectional and serve to transmit knowledge and support [1,6].

### Social Support and Type 2 Diabetes Self-Management

A community of like individuals fosters the continued exchange of information and support, which may help an individual with chronic illness to not feel alone while engaging in chronic illness self-management [7-12]. Individuals with type 2 diabetes (T2D) complete the majority of self-management outside of a health care setting [13] and struggle to adopt healthy behaviors [14-16]. The frequency and amount of T2D self-management behaviors may cause an individual to feel overwhelmed, which may lead to further feelings of frustration and general inattention to these self-management behaviors [17,18]. Notably, individuals with T2D benefit from frequent and sustained interactions with providers and peers (eg, others with T2D) because they obtain critical self-management education and support [19-21]. Yet, frequent interaction with providers and peers in face-to-face environments may not be feasible because of temporal, financial, and geographical limitations [18,22-24].

### Synchronous Virtual Environments Facilitate Social Interaction and Support

One way to provide personalized, frequent interaction and support is via T2D-specific VEs that assist individuals in health care decision making [10-12,25]. Research indicates that these VEs facilitated the interaction among providers and peers and replicated locations that an individual with T2D would typically encounter while engaging in self-management [10-12,25]. However, these studies provided little information on how these VE-mediated interactions among peers and providers compared with face-to-face interactions. Therefore, an understanding of *how individuals interact* in a disease-specific VE and *what these individuals discuss* can improve how researchers design consumer health informatics interventions aimed at improving self-management. Additionally, an examination of *how the VE mediates the interaction* is needed to understand the extent to which VEs, and similar types of consumer health informatics media, can supplement traditional face-to-face encounters with providers and peers who support chronic illness self-management. Thus, the purpose of this study was to describe social interaction and support among individuals with T2D who interacted via a VE to obtain T2D-specific education and support.

## Methods

We briefly describe the research approach below; a detailed protocol has been described elsewhere [26]. The Duke University Institutional Review Board (Pro00022132) approved this secondary analysis. We did not collect any new data or recontact the participants.

### Guiding Framework

**Figure 1:**
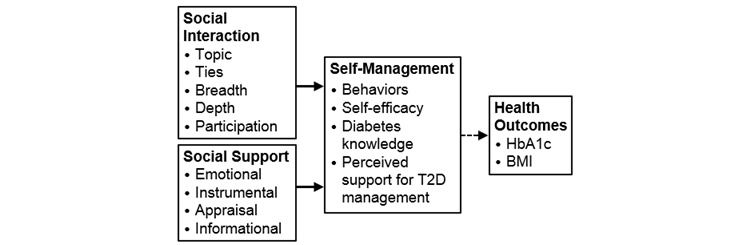
Guiding framework for this secondary analysis. T2D: type 2 diabetes; BMI: body mass index; HbA_1c_: glycated hemoglobin. This figure was originally published in Lewinski AA et al [26].

A guiding framework based upon social penetration theory [27] and strong/weak tie theory [28,29] guided the description of social interaction and support in a T2D-specific VE. The guiding framework (Figure 1) [26] includes the concepts *social interaction*, *social support*, *self-management*, and *health outcomes*; this paper addresses the concepts of interaction and support.

### Parent Study and Sample

Data for this secondary analysis came from the Second Life Impacts Diabetes Education & Self-Management (SLIDES) (1R21-LM010727-01) study. Information on the SLIDES study site, sample, measures, and outcomes is published elsewhere [12,30]. The SLIDES study provided 2 weekly T2D education sessions and 1 weekly support session via a VE hosted on Second Life for adults with T2D [12,30]. The total sample for this analysis (N=24) included study participants (n=20) and investigators and diabetes educators (n=4). The study participant demographics were as follows: women (19/20, 95%), male (1/20, 5%); mean age of 54 years; white (13/20, 65%) , black (7/20, 35%); married (11/20, 55%); with an annual income of US $50,000 or greater (14/20, 70%); with a bachelor’s degree or higher (13/20, 65%); and all of them were regular users of the Internet [12,30]. The SLIDES site was password protected, and participants chose anonymous screen names.

### Qualitative Data

**Figure 2:**
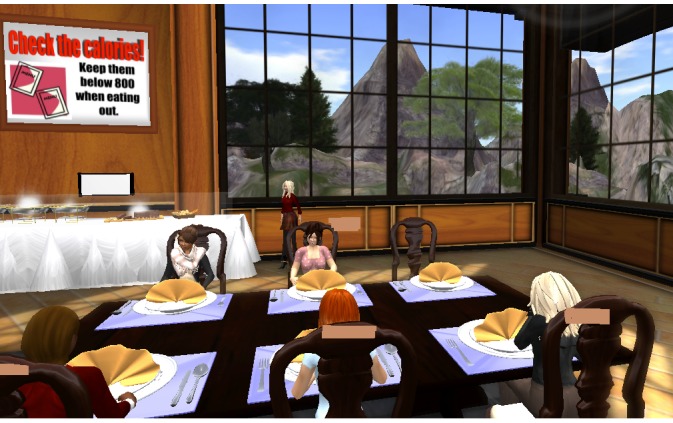
Synchronous support session in the restaurant in the virtual environment. The avatars of the individuals living with type 2 diabetes and the diabetes educator are sitting at the restaurant table and discussing healthy food options.

**Figure 3:**
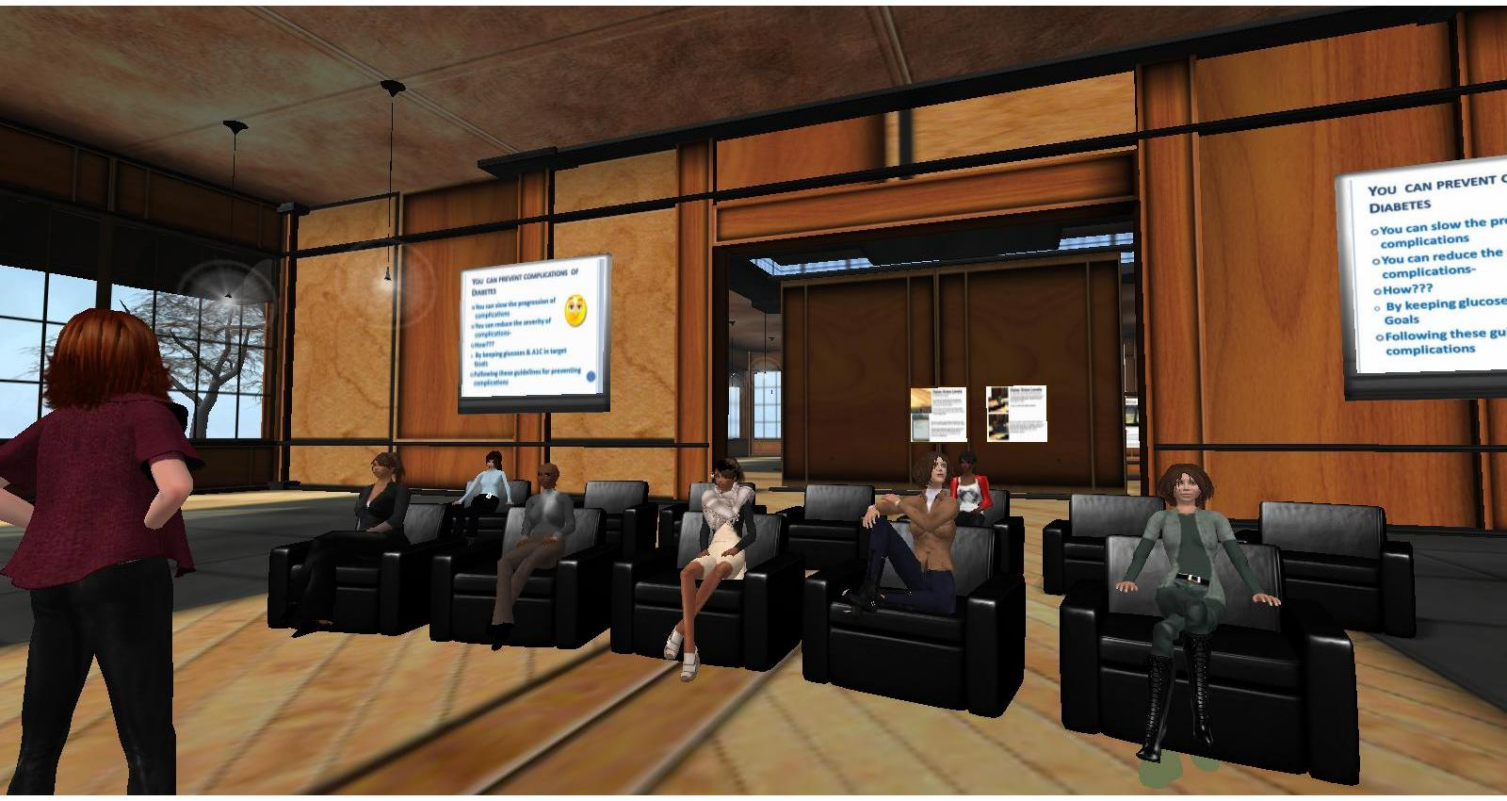
Synchronous education session in the community center in the virtual environment. The diabetes educator’s avatar is standing and leading the class, the avatars of the individuals living with type 2 diabetes are sitting in the chairs, and the session PowerPoint slides are visible on the large screens on the right and left.

Qualitative data included transcribed real-time conversations, emails, discussion board postings, and text-chat transcripts among participants and between participants and diabetes educators within the VE over each participant’s 6-month study enrollment. Most data included synchronous conversations that occurred when participants and diabetes educators interacted and talked with each other as avatars while in the VE. Figure 2 depicts a synchronous support session in the restaurant, and Figure 3 depicts a synchronous education session in the community center. Participants did not use the text-chat or discussion boards frequently to engage with other participants or the diabetes educators. We imported transcribed conversations into Atlas.ti version 7.5.17 (Atlas.ti GmBH, Berlin, Germany) for analysis.

### Analysis

We used content analysis to describe social interaction and support among participants who engaged in a T2D-specific VE [31,32]. We modified the first- and second-level coding procedures as previously explained [26] to describe *how individuals interacted* (eg, verbal techniques), *what was said* (eg, topic), and *how the VE mediated this interaction* [33].

A coding team comprising 3 authors (AAL, RAA, and CMJ) developed codes based on the guiding framework. The coding team met biweekly and discussed and reviewed all codes and emerging themes until they reached a consensus. In these meetings, the coding team ensured that all codes were richly defined, the codebook included appropriate exemplar quotations, and the codes were consistently applied to these data [33-35]. Interrater reliability may invalidate research with unstructured qualitative data [35]; therefore, the coding team coded these conversational data by consensus to ensure reliability and validity of the codes and coded segments [33-35]. The first author (AAL) independently coded a segment of data, and then the second (RAA) and last (CMJ) authors independently reviewed the first author’s coding. When the coding team disagreed, the original coding would remain if the rationale for the coding was clearly articulated and included an audit trail, even if the other coders had additional interpretations. The coding team revised coding only when the secondary coders deemed the rationale for the coding as not credible. In those cases, the coding team worked to reach an agreement about the appropriate code and revised prior coding as appropriate using the new rationale. The coding team resolved disagreements through extensive discussion, which ensured the reliability and validity of the interpretation of these data [33-35]. This process was repeated for all first- and second-level codes. In total, the second and last authors each independently reviewed 25% of the first author’s coding.

## Results

In the following sections, we describe how participants interacted in the VE and the support participants exchanged in the VE, with examples of some of the essential features identified and listed in the tables. In the findings, unless otherwise specified, the term *participant* refers to both an individual with T2D and a diabetes educator.

### Social Interaction: How Participants Interacted in the Virtual Environment

VE-mediated interactions consist of the following: (1) communication techniques (how participants interact in real-time communication in a VE); (2) expressions of self-management (the content of participants’ self-management discussions); (3) depth of conversation (intensity of information shared); and (4) breadth of conversation (the number of topics discussed among participants in a conversation).

#### Characteristic #1: Communication Techniques

Participants used 4 types of communication techniques when they interacted and conversed with other participants. These include indicators of listening, being in the VE, attributes of bidirectional information exchange, and connecting actions. Table 1 details these types and provides a definition of the interaction behaviors (ie, participant actions during an interaction) and exemplar quotes or instances.

**Table 1:** Communication techniques.

Interaction behaviors and definition			Exemplar quote or instance
**Indicators of listening—verbal utterances that indicate that someone was present and listening**			
	Double-checking (clarification of a term, idea, or statement)		A participant stated how she felt at certain glycemic values. The diabetes educator (DE) clarified the values and her actions.
	Following conversation (indication a person is listening to the conversation)		“Uh huh.”
			“Mmmhmm.”
	Reflecting back (instances in which a person talking reflects back something someone else has stated)		When the DE heard some participants do not consume alcohol, she stated, “Ok, well that’s good. I’m glad to hear that.”
	Repeating phrase (repeating a phrase when asked to repeat the phrase)		Participants repeated questions or comments to obtain information.
	Responsiveness (instances of positive feedback during interactions)		During a lecture, the DE asked, “I heard a squeak. Somebody say something?”
	Nonresponsiveness (dismissing a question or comment in an interaction)		Instances included when a participant’s question or comment was not acknowledged.
	Being busy (statements of how a participant is busy with life events)		“I had a real important meeting. I had to go to.”
			A time mix-up occurred and prevented an activity.
	Interrupting another (interrupting a conversation or talking over someone)		Instances when the speaker’s sentence is cut off by another individual’s verbal utterance.
	Inappropriate comment (offensive words)		“Dorks.”
			“Nerds.”
**Being in the virtual environment (VE)—indications that participants felt they were in the VE and they were not alone**			
	Feeling presence (the influence of the VE on interaction)		“Wait a minute. Where am I [avatar] taking off to?”
			“These [items in the grocery store]? Oh I’m pointing on my screen [with my hands], how handy is that?”
	Feeling VE copresence (indications a participant is in the VE with others)		“I’m getting tired of standing behind this podium because I never do this in the real world anyway.”
	Practicing self-management skills (practicing self-management skills in the VE)		“Here we have a simple label. For this particular food, can anybody read what the serving size is?”
	Stating location (statements of what is occurring in the VE)		“Everybody’s outside right now. Come on outside. We’re out near the [location].”
**Attributes of bidirectional information exchange—statements used to exchange information**			
	**Posing**		
		Seeking information (asking a question for more information)	Participants asked questions about self-management topics covered in the education and support sessions.
	**Responding**		
		Giving a reminder (reminding an individual about something)	“Remember we said one piece of bread equals one starch serving.”
		Giving information (giving content about type 2 diabetes (T2D) self-management)	When participants provided information on T2D self-management during sessions.
		Answering a question (the act of responding)	“In reply to your question last week...”
			“Let me answer your question.”
		Correcting someone or oneself (correcting someone about T2D self-management)	“Nope. That’s not right.”
			“No, because fat is not a starch.”
		Adjacency pair (question-and-answer pair)	Instances in which there was a direct and immediate response.
**Connecting actions—statements and actions taken by individuals when associating with others**			
	**Personality attributes**		
		Being engaged (an interest or opinion about T2D self-management)	A participant asked, “Will the topic be the same for the other session if I end up going during the day?”
		Being encouraging (encouragement for self-management behaviors)	“Wow! That’s good.”
			“Great job!”
		Being enthusiastic (excitement)	“Oh good!” or “Great!”
		Being friendly (wishing someone well or being nice to a person)	“I hope you are doing well.”
			“Don’t worry, you are doing fine.”
		Being incredulous (laughing or having an awkward response)	During a session, participants stated that they did not drink alcohol. The DE responded with “Really!”
		Being polite (polite phrases)	“Thank you” or “Please.”
		Collaborating (working together to solve a problem)	“Let’s look at this meal together and see if we can make it more diabetes friendly.”
		Commiserating (admitting that problems happen to everyone, and people are not alone)	In response to a participant trying to drink more water, a DE said, “Yeah. It definitely is an adjustment. It takes a lot to get used to it.”
		Expressing concern (concern about someone)	“You sound terrible tonight! How are you feeling?”
		Expressing empathy (showing empathy for another participant)	“Oh, I’m sorry about that.”
		Expressing gratitude (expression of thanks for an effort during the session)	“Thank you for participating tonight.”
		Helpfulness (instances of helpful actions)	A participant repeated what someone said in response to a third participant stating “I couldn’t hear her clearly.”
	**Signs of copresence**		
		Calling by name^a^ (addressing someone by their avatar name)	“Hi [name].”
			“Good question [name].”
		Commenting on appearance (comments on avatar’s appearance)	“Do you have another new outfit on?”
			“What a lovely shirt you have on.”
		Greeting (saying a variation of hello or good-bye)	“Hello” or “Good bye.”
			“Hi! How are you?”
		Introducing oneself (stating their name and role)	“My name is [name] and I have had diabetes for [years].”
		Noticing others (noticing if another participant is present or absent)	“And [name] was talking, and she stopped with [name] when we started to walk over.”
	**Moderating the conversation**		
		Checking-in (seeing if anyone has any questions)	“Before we go any further, do you have any questions from last week’s session?”
		Connecting outside the VE (interactions outside the VE)	“Call me in the office tomorrow.”
			“Let’s talk at your next appointment.”
		Facilitating interaction (connecting participants together)	“What are the most challenging issues for you in terms of diabetes nutrition?”
		Referring to shared history (discussing a shared history or knowledge between themselves)	“How did everything go at the doctor this week?”
			“We talked about this in the first class that the reason why…”
		Sticking to time schedule (stating that someone is on a time schedule)	“It’s about 5 after, so I’ll get started.”
			“I don’t want to keep you if you need to go.”

^a^Name refers to screen name.

##### Indicators of Listening

Indicators of listening promoted or inhibited subsequent interactions among individuals because of how these behaviors influenced the exchange of information and support. For example, participants exhibited *responsiveness*, a behavior that promoted interaction, when they responded to questions or engaged with others. Conversely, there were instances of *nonresponsiveness*, a behavior that inhibited interaction. For example, the diabetes educator exhibited *nonresponsiveness* when they were speaking, and a participant interrupted with a question to which the educator did not respond. However, *nonresponsiveness* may have occurred due to inherent features of the VE, such as the lack of nonverbal cues (eg, one cannot visually see when another participant opens their mouth to speak) or problems with technological equipment (eg, broken headsets).

##### Being in the Virtual Environment

Participants indicated feelings of being in the VE when they used the embedded components (eg, grocery items) of the VE to interact with other participants as avatars. These embedded components (eg, restaurant menus) facilitated interaction because they stimulated conversation and extended the interaction among participants. During the interactions, the position of another participant’s avatar during the exchange of information and support also mattered. For example, the behavior *stating location* promoted interaction because it enabled participants to colocate others in the VE. Participants used statements such as “She’s standing right behind you” or “I’m standing right next to you” when they colocated each other in the VE. The position of a participant’s avatar served as a proxy for their inclusion in the interaction, and in turn, the subsequent discussion offered participants the opportunity to share and obtain self-management information and support.

##### Attributes of Bidirectional Information Exchange

Participants used several interaction behaviors when they exchanged information. The behaviors *posing* (ie, asking a question) and *responding* captured the bidirectional nature of an interaction. Prompt responses promoted further interaction, because the participant who asked the question immediately received relevant information. In several instances, participant responses segued into new topics, introduced new T2D content, or summarized the topics reviewed in the session.

##### Connecting Actions

Participants used connecting actions in the VE that resembled interaction behaviors in face-to-face environments. The 3 types of *connecting actions* are as follows: (1) personality attributes— the participant’s personal characteristics that became evident during interactions; (2) signs of copresence— techniques that alerted others to one’s presence in the VE; and (3) moderating the conversation— actions of the diabetes educators during the sessions. Participants were helpful when they included others in the conversation, called each other by avatar name, or provided information about self-management. Importantly, the diabetes educators looked to see which participants signed into the VE at the beginning of each session and monitored the presence of participants during the session.

#### Characteristic #2: Expressions of Self-Management Behaviors

We identified 2 types of expressions of self-management behaviors in a social interaction: (1) challenging aspects of T2D and (2) self-managing in the real world. Each is described with examples of some of the essential behaviors that were identified in Table 2.

##### Challenging Aspects of T2D

Participants stated limitations and problems, mistakes, and psychosocial aspects, which increased the self-management effort required. However, when participants stated their limitations during the sessions, they also sought information on ways to ameliorate the problem (eg, trying water aerobics instead of weight-bearing exercises). Participants revealed a myriad of challenges in unprompted statements; these admissions of difficulty with self-management evolved from discussions during the sessions.

##### Self-Managing in the Real World

Participants discussed coping (eg, problem solving), their self-management intentions and objectives (eg, self-management choices), and the external influencers (eg, how others influenced their self-management) in sessions. During discussions, participants shared real-world practices as to how they identified successful self-management strategies. For example, one participant stated she felt overwhelmed with her T2D diagnosis and other comorbidities. In response, several other participants shared how they coped with feeling overwhelmed. Participants shared positive changes in their health status, explained how they met a self-management goal, or described a plan to meet a self-management objective during the sessions.

#### Characteristic #3: Depth

We operationalized the concept *depth* as the degree of personalized information shared in a social interaction in the VE. The levels of depth occur on a continuum, where level 1 indicated little to no personalization of information shared by the participant, and level 4 indicated that the participant shared highly personal information and acknowledged weaknesses related to T2D self-management [27,36,37]. Table 3 provides the definitions of each level of depth.

Participants revealed personal information when they asked topic-relevant questions during a session or when they gave information or support to another participant. Participants also revealed difficulties with certain self-management behaviors and referred to past instances to highlight mistakes and problem solving. The majority of the diabetes educators’ personalized statements related to the topic being discussed by the participants, and these statements were used to stress a point or provide reassurance. Notably, when one participant shared personal information, another participant followed up with his or her own experiences providing support and information. Participants answered personal questions when they discussed their struggles with T2D self-management; participants did this to help others or obtain support for themselves.

#### Characteristic #4: Breadth

The conversations in these data concerned topics related to T2D self-management (eg, nutrition, foot care) and overall health. Most discussions aligned with the weekly focus of the education sessions. These findings indicated that there was no significant variance in subjects outside of T2D-related topics.

**Table 2:** Description of expressions of self-management.

Interaction behaviors and definition					Exemplar quote or instance
**Challenging aspects of living with type 2 diabetes (T2D)—statements of challenges in T2D self-management**					
	**Stating limitations and problems**				
		Stating limitations (a stated financial, temporal, physical, or geographic limitation)			“I rarely go out to eat because it is just too much effort for me. It would be nice to leave my apartment, get out of the house, and drive to the drive-in. My daughter told me that Wendy’s has salads. Just to get out of the house and not so home-bound all the time.”
		Lacking health knowledge (lack of T2D knowledge)			In the virtual environment (VE) grocery store, a participant stated: “Well, I looked at the regular yogurt versus the Greek yogurt ’cause I eat light yogurt, and I was surprised at how much sodium it had in it. It’s not a lot as far as the number, but I thought it wouldn’t have any sodium in it.”
		Stating problems (a problem related to self-management)			A participant’s daughter buys the participant unhealthy foods or foods the participant does not like.
		Admitting difficulty (difficulty applying concepts related to self-management)			“I’m having a hard time drinking my water.”
					After receiving a compliment on her weight loss, a participant stated, “It’s great though, very tough, but it can be done.”
	**Mistakes**				
		Making self-management mistake (admitting to a mistake when doing self-management)			“When I first started, I was told to wash my hands. I was diagnosed in [date] and you get sloppy over the years. I had not washed my hands or anything like that. Then I had another [apple] as a snack so when it came supper you can imagine what was on my finger. I had this 379 for suppertime sugar [the individual’s blood glucose reading on her glucometer prior to eating supper was 379 which was much higher for her than normal].”
	**Psychosocial aspects**				
		Frustration (expressing frustration about self-management)			“That was a new experience for me. That they [insurance company] can change the meter that you use. I didn’t like that.”
		Feeling isolated (feelings of social isolation due to physical limitations or living with T2D)			“I’m the only one in my family with diabetes. Nobody has ever been around anyone with diabetes before in our family. My grandmother had it but she passed on and so they don’t understand what I am going through and what they need to do to help me.”
**Self-managing in the real world—statements related to enacting T2D self-management**					
	**Coping**				
			Satisficing (choosing a self-management option that is the best choice within the available options)		When discussing a T2D-friendly menu item, a participant stated she goes to fast-food places because she has limited mobility and they have drive-thrus. She described how she worked with her dietitian to identify healthy items at the fast-food restaurants.	
		Problem solving (a behavior one engages in to accommodate a physical, financial, temporal, or geographical barrier to engage in T2D self-management)			T2D and depression: “I laugh a lot. I don’t have to tell you I laugh. I laugh a lot every day and it’s really healing to you. It is. Makes you feel better.”
					“I went to [restaurant], and my friend has an app that can tell you the ingredients. I was completely surprised at what I thought was a relatively good choice of this 1/2 salad and something else. [That experience] helped me realize I need to look ahead.”
	**Self-management intentions and objectives**				
		Stating self-management behavior or making a self-management choice (a statement of how a person completes a T2D self-management behavior)			“I use a lot of herbs and spices, and I’m trying to cut back on using a lot of salt.”
					“My husband brought me some sugar-free candy for [holiday] last year and I said why did you bring me sugar-free candy? He said because that is what you need! And I said okay, thank you. But I really don’t eat candy. I’m gonna avoid candy tomorrow [holiday]. I don’t want any candy.”
		Demonstrating knowledge (when a participant is knowledgeable about T2D self-management			When looking at items in the restaurant, a participant stated, “...it is the sodium that is not bad, but you are getting a whole meal plus of carbohydrates!”
					Saying, “A smaller serving size of the cereal” when modifying a meal in a session.
		Self-efficacy for self-management (when a participant states he or she can do something related to T2D self-management)			“I’ve been real good [about preventing eye complications]. I’m going to get my surgery done and I am going to be okay. I am not going to give up.”
					Dealing with an unhelpful family member, a participant stated, “I just tell him to get off my back. That I’m doing the best I can.”
	**External influencers**				
		Referring to family or friends (mentioning friends and family while in the VE)			“Yeah, I was diagnosed with diabetes this year. This is new to me but I have a brother who had diabetes and he passed away in [date] from diabetes-related circumstances and situations. So this runs in the family. My mother had diabetes, and so it’s in the family.”
		External social environment (social instances and T2D self-management)			“I was reading a magazine while waiting to get my tires rotated. They had a brown rice diet from [University]. I just want to know, what you [diabetes educator] thought about that.”

**Table 3:** Description of depth. Definition based upon social penetration theory and related literature.

Attribute and definition	Exemplar quotes
Level 1: Making small talk (no personal health information revealed)	“I was out last week, out of town and I didn’t have Internet access.”
	“I had a new grandbaby arrive.”
Level 2: Opening up (hinted at personal issues and shared observations about others)	“What if you have other limitations? Like if you are on a walker or something like that? Man, some of those exercises are not going to very well work for you.”
Level 3: Informing (shared objective facts about type 2 diabetes)	“Okay, ’cause when I saw you a month ago, it was 6.2 [the participant’s hemoglobin A_1c_ value], and I wondered what if I didn’t have diabetes?”
Level 4: Disclosing (highest amount of personalization, revealed weaknesses)	“I had major surgery about a year and a half ago that brought my life to a standstill. I am pretty much, well I am homebound except, my big social life is when I go to the doctor’s office.”

##### Social Support: The Support Participants Exchanged in the Virtual Environment

Participants exchanged support in the VE in the twice-weekly education and weekly support sessions. A total of 1170 support exchanges occurred in the education (723/1170, 61.79%) and support (406/1170, 34.70%) sessions, or outside of session times (eg, before or after each session; 41/1170, 3.50%). The 4 types of support were not exchanged in equal measure, as informational (535/1170, 45.73%) was the most exchanged, followed by emotional (377/1170, 32.22%) support. Few instances of appraisal (217/1170, 18.55%) and even fewer instances of instrumental (41/1170, 3.50%) support were noted in these data.

##### Emotional Support

Emotional support includes feelings of empathy, trust, and caring. Diabetes educators and participants exchanged 3 types of emotional support in real-time conversation: (1) physical health (88/377, 23.3%) or empathy for the physical challenges and symptoms of T2D; (2) psychosocial (189/377, 50.1%) or empathy for psychosocial aspects (eg, loneliness, depression, frustration) when one lives with T2D; and (3) motivational (100/377, 26.5%) or empathy and encouragement for engaging in T2D self-management behaviors.

Emotional support interactions centered on integrating T2D self-management into one’s daily life. Participants elicited and provided emotional support when they discussed the physical challenges (eg, hypoglycemic events), the psychosocial issues (eg, loneliness), and restrictions (eg, unable to eat certain foods) encountered when living with T2D. The statements that elicited emotional support included participant responses to questions during the education lectures, participant responses to direct questions from a diabetes educator or participant, participant comments during a discussion, or a participant-initiated statement. Supportive comments included short phrases such as “Oh no,” and “Mmmhmm,” or longer phrases such as “Well, that is good news” and “I knew I wasn’t the only one!” Typically, these phrases occurred simultaneously while the participant (the support elicitor) revealed personal information or immediately after the participant concluded his or her statement.

##### Informational Support

Diabetes educators and participants frequently exchanged informational support, or T2D-specific information, in the VE. In total, we noted 10 types of informational support. There were 8 types of informational support that correlated with the predetermined session topics: nutrition and food (149/535, 27.9%), preventing complications and problem solving (85/535, 15.9%), monitoring (55/535, 10.3%), medications (insulin; 36/535, 6.7%), medications (not insulin; 35/535, 6.5%), psychosocial aspects and coping (29/535, 5.4%), pathophysiology (28/535, 5.2%), and exercise (24/535, 4.4%). Additionally, we noted that participants exchanged 2 types of informational support that related to the SLIDES study (81/535, 15.1%) and on miscellaneous topics (13/535, 2.4%; eg, information on local places).

Participant-initiated questions and comments stimulated the conversation and subsequent sharing of information by the diabetes educators or the other participants. Informational support was elicited through questions or concerns raised during the discussion. These elicitation behaviors enabled the diabetes educators and the other participants to respond with informational support. For example, participants posed questions such as “What about sugar-free sodas?,” “What is [drug]?,” or made statements such as sharing one’s personal exercise routine. These instances resulted in informational support in the form of suggestions such as quick and easy breakfast choices, ideas for healthy beverages, and how to incorporate more water into one’s diet. We noted few instances in which the diabetes educators or individuals delivered information and/or support via corrective feedback to address misinformation about T2D self-management knowledge and behaviors. Overall, the informational support cleared up misunderstandings and enabled each participant who initiated an exchange to ask questions until the topic was clear.

##### Appraisal Support

Appraisal support, or affirmational statements regarding information seeking and engagement in T2D self-management, was exchanged by everyone in the VE. The 2 types of appraisal support are (1) support for information seeking (32/217, 14.7%) or an affirmational reply after a participant asked a question or provided a comment, and (2) support for self-management behaviors (185/217, 85.3%) or an affirmational reply following a participant’s statement about a specific T2D self-management behavior. Diabetes educators affirmed the participant’s information-seeking behaviors when they positively responded (eg, “That is an excellent question”) during classes or when the participants shared personal information. Diabetes educators and participants congratulated and praised other participants when they discussed how they overcame a challenge, engaged in a preventative behavior, or shared positive news about themselves. For example, when a participant stated how much money she spent on her eye problems, someone stated “Yeah, that is important, stay on top of your eye problems,” which reaffirmed the participant’s self-management behaviors.

##### Instrumental Support

Instrumental support, or the exchange of tangible goods related to T2D self-management, was the least exchanged type of support in the VE. Instrumental support exchanges occurred when participants exchanged website links and information (31/41, 76%), information on specific self-management tools (7/41, 17%), and recipes (3/41, 7%). Participants provided instrumental support following a discussion in an education or support session (eg, links to understand a T2D symptom) or in response to a question (eg, questions for meal ideas).

### Contributions of Virtual Environment–Facilitated Education and Support Sessions

**Figure 4:**
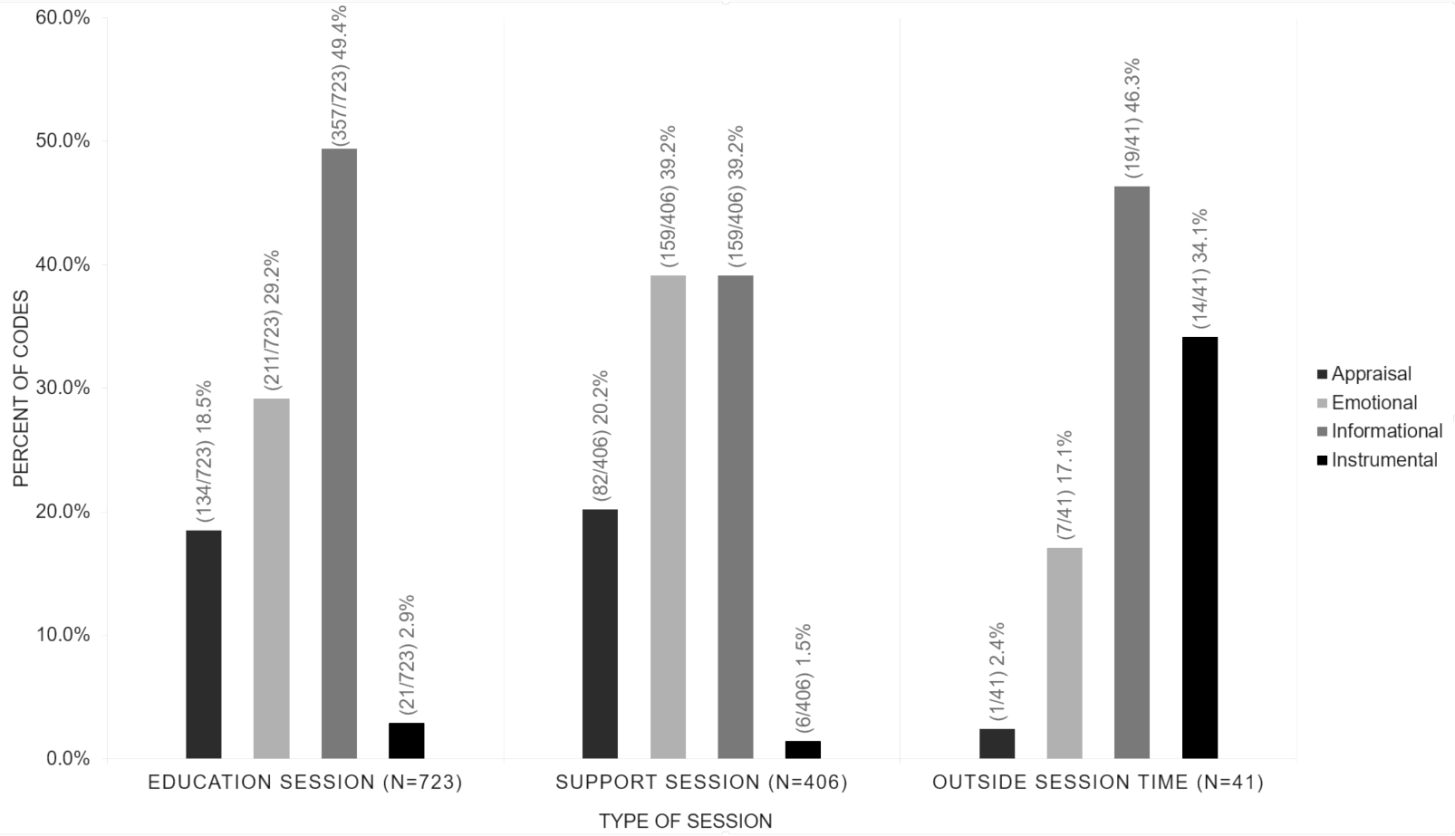
Frequency of each type of social support as exchanged in the education and social support sessions, or outside of session time (eg, before or after each session).

We disaggregated the 4 types of social support by education and support session to determine when the social support was exchanged (see Figure 4).

The education sessions provided predominately informational support (49.4%, 357 of the 723 exchanges). One way the diabetes educators provided informational support was during the lectures on prespecified content; lectures included PowerPoint slides, learning activities, and questions to facilitate discussion. The general support sessions provided predominately emotional (39.2%, 159 of the 406 exchanges) and informational (39.2%, 159 of the 406 exchanges) support. One way the participants provided emotional support was through the sharing of their own self-management experiences in response to questions from the diabetes educator or other participants. Overall, the discussion during the sessions enabled participants and diabetes educators to converse about current self-management behaviors and challenges.

## Discussion

### Principal Findings

We described how participants interacted and exchanged support in a VE. Specifically, we showed that individuals with T2D discussed personal information (ie, ties, depth, participation), that there was not great variation in topics in a moderated conversation (ie, breadth), and also described the content of a supportive interaction in the VE. Together, these data indicate that VE–mediated interactions are similar to interactions in face-to-face environments. Individuals interacted in similar ways to interactions in a face-to-face support group when they shared personal information to obtain education and support for their own T2D self-management behaviors.

### Interaction in a Virtual Environment

VE interactions are multidimensional because the VE mediates the communication techniques (eg, indicators of listening) individuals use when talking among themselves to form relationships with each other. Similar to face-to-face interactions, individuals in the SLIDES study used techniques such as greeting, responding to questions, and noticing others to indicate their engagement in the bidirectional interaction. These communication techniques resemble the cues present in a face-to-face interaction that facilitates the exchange of information, in addition to the individual’s emotions and feelings [38-43].

Appearance is equally important in the VE as in face-to-face environments. Avatars that look like, and behave like, real humans influence feelings that one is in the VE with others; this increased awareness and acknowledgment of others in the VE may help the development of a community in which repeated interactions occur [1,3,44]. The individuals in our study looked for the avatars of other individuals and made statements such as “I’m standing behind you,” and “I’m over here” when individuals wanted to see each other. These statements indicated participants felt *presence* and *copresence* in the VE. Our results provide insight into, and further describe, the finding that avatars serve as a proxy for others in the VE [45].

Individuals entered into the VE as weak ties to others, to obtain T2D-specific information and personalized support from diabetes educators and other individuals with T2D. Weak ties do not require a large amount of investment, the tie can be formed rapidly, and the tie brings in novel information to a group of individuals [28,29,46]. Examples of weak ties in the VE occurred when individuals shared information during the education and support sessions and provided personalized information when they responded to other individuals’ or educator’s questions, or provided a comment about their self-management experience. Overall, the findings are parallel to, but not equivalent to, Granovetter’s strong/weak tie theory [28,29]. Just as the weak ties outside a tight-knit group can provide useful information to the group, our findings show that useful information is also exchanged among a group of people with whom the individual has ties of varying strength.

The repeated interactions (ie, over time, sharing personal information) among individuals in the VE may have facilitated the progression of weak ties into strong ties. Similar to face-to-face interactions [47,48], the individuals in our study discussed varying amounts of personal information with each other in response to questions and comments. Over time, we observed that individuals referenced previously discussed personal information when they responded or engaged with another individual; this may be a sign that time, and depth of conversation, contributes to tie formation among individuals. In contrast, an individual who did not regularly attend sessions, share personal information, or verbally participate did not build relationships with other individuals in the VE that facilitated the transition from a weak to a strong tie.

A strong tie exists between individuals when there is increased frequency, duration, and closeness of contact, and a direct link between 2 individuals [28,29,46]. Frequent and positive interaction with providers (eg, diabetes educators), or a strong tie connection, positively influences health behaviors and health outcomes for individuals with T2D [49]. In the VE, the diabetes educators interacted with participants during the sessions; we noted that these interactions were bidirectional as both the diabetes educator and the participant provided personal information about themselves. This style of interaction is similar to what occurs in face-to-face support groups, where diabetes educators serve as facilitators by connecting others, exchanging information, managing group dynamics, and prompting problem solving [50]. These actions created a relaxed learning environment, which resulted in a safe space in the VE where an individual could disclose his or her personal challenges and obtain personal support [49,50].

Individuals who interact online may do so because the Internet enables dialogue between peers and providers about sensitive information while remaining anonymous [51-53]. Although the diabetes educators in the SLIDES study used their real names, the study individuals each had an anonymous screen name and customized their avatar to their personal preference [12]. These features may have helped individuals to share personal information [10,54,55] and potentially more information than in a face-to-face interaction [56].

### Support in a Virtual Environment

Similar to a face-to-face interaction, the VE enabled individuals to promptly elicit support and provide supportive responses; these timely exchanges may have resulted in increased feelings of support and the desire to reciprocate the support received [57,58]. However, the VE facilitated routine, positive, interactions with peers and providers from the comfort of one’s own home. The interactions with peers who are successful and unsuccessful in T2D self-management enable individuals to problem-solve and identify ways to address their own challenges [8,47,59]. These exchanges prompted further discussion and opportunities to create supportive relationships among a diverse group of peers [47,60], which further substantiates the SLIDES study result that showed a statistically significant increase in social support from the beginning to the end of the study [12]. Overall, our results are similar to face-to-face interactions in which individuals discuss T2D failures and successes to exchange support [47,48,61].

### Limitations

This research has several limitations. Due to the small size of the sample (N=24) and only 1 male participant, these findings should be interpreted with caution. We did not analyze each individual’s participation over time in the VE, as this type of analysis is beyond the scope of this descriptive study. As participants entered into the SLIDES study at various time points, the most accurate way to assess tie development, tie strength, and amount/type of support each participant exchanged would be to analyze each participant’s interactions with the other participants. Future research should use a case study approach to analyze interaction and support at an individual level to determine how interaction, tie development and strength, and support differ by participant (eg, gender, participation). Despite these limitations, the findings are valuable because of the insight provided on social interaction and support in a VE.

### Conclusions

In this descriptive study, we analyzed conversations among adults who interacted in a VE. We described *how individuals interacted*, *what these individuals discussed*, and *how the VE mediated the interaction*. Our data indicate that the realistic VE, in conjunction with the ability to synchronously interact with others, may have accurately replicated face-to-face interactions. VEs, a type of consumer health information technology media, may provide individuals a full range of interaction that includes informational, emotional, and appraisal support. With this versatility, VEs are able to contribute substantially to the support for those with T2D and very likely other chronic illnesses.

## Abbreviations

DE: diabetes educator
SLIDES: Second Life Impacts Diabetes Education & Self-Management
T2D: type 2 diabetes
VE: virtual environment
